# Targeting Determinants of Dosage Compensation in *Drosophila*

**DOI:** 10.1371/journal.pgen.0020005

**Published:** 2006-02-03

**Authors:** Ina K Dahlsveen, Gregor D Gilfillan, Vladimir I Shelest, Rosemarie Lamm, Peter B Becker

**Affiliations:** 1 Adolf-Butenandt-Institut, Molekularbiologie, Ludwig-Maximilians-Universität München, München, Germany; 2 Institute of Laser Physics, Siberian Branch of RAS, Novosibirsk, Russia; Stowers Institute for Medical Research, United States of America

## Abstract

The dosage compensation complex (DCC) in Drosophila melanogaster is responsible for up-regulating transcription from the single male X chromosome to equal the transcription from the two X chromosomes in females. Visualization of the DCC, a large ribonucleoprotein complex, on male larval polytene chromosomes reveals that the complex binds selectively to many interbands on the X chromosome. The targeting of the DCC is thought to be in part determined by DNA sequences that are enriched on the X. So far, lack of knowledge about DCC binding sites has prevented the identification of sequence determinants. Only three binding sites have been identified to date, but analysis of their DNA sequence did not allow the prediction of further binding sites. We have used chromatin immunoprecipitation to identify a number of new DCC binding fragments and characterized them in vivo by visualizing DCC binding to autosomal insertions of these fragments, and we have demonstrated that they possess a wide range of potential to recruit the DCC. By varying the in vivo concentration of the DCC, we provide evidence that this range of recruitment potential is due to differences in affinity of the complex to these sites. We were also able to establish that DCC binding to ectopic high-affinity sites can allow nearby low-affinity sites to recruit the complex. Using the sequences of the newly identified and previously characterized binding fragments, we have uncovered a number of short sequence motifs, which in combination may contribute to DCC recruitment. Our findings suggest that the DCC is recruited to the X via a number of binding sites of decreasing affinities, and that the presence of high- and moderate-affinity sites on the X may ensure that lower-affinity sites are occupied in a context-dependent manner. Our bioinformatics analysis suggests that DCC binding sites may be composed of variable combinations of degenerate motifs.

## Synopsis

In fruit flies, just like in humans, the two sexes are distinguished by different sex chromosomes. Females have two X chromosomes and hence a double dose of all X-linked genes when compared to males, which only have a single X chromosome. This different gene dosage needs to be compensated for by adjusting transcription levels such that male and female cells synthesize equal amounts of gene products. In *Drosophila melanogaster,* dosage compensation occurs by doubling the transcription of many genes on the single male X. This chromosome-wide control is achieved by a male-specific dosage compensation complex (DCC), which contains enzymes, structural proteins, and non-coding RNA. How is the DCC able to distinguish the X chromosome from the autosomes for selective interaction? In the following article, the authors identify and characterize several novel DNA sequences on the X chromosome that can recruit the DCC. Their results suggest that the X chromosome contains a large number of binding sites for the DCC, which are made up of combinations of degenerate sequence elements. These elements constitute binding sites with varying affinities for the complex. Collectively, their abundance on the X chromosome restricts the action of DCC to the X chromosomal territory.

## Introduction

A mechanism for selecting and marking an entire chromosome for coordinate regulation is central to the process of dosage compensation. Genetic sex determination in animals usually involves a pair of heterologous sex chromosomes and leads to a chromosome imbalance between males and females. Consequently, the process of dosage compensation is necessary to ensure equal expression of sex chromosome–linked genes. Studies in different species have revealed that dosage compensation may be achieved through different modes of chromosome-wide transcriptional regulation, and that different means of targeting the selected chromosome may be employed (reviewed in [1]). In all cases, the chromosome or chromosomes to be regulated are marked by the presence of specific protein or ribonucleoprotein complexes as well as alterations in chromatin structure.

In mammals, dosage compensation is achieved by the inactivation of one of the two X chromosomes in females. The selection of this chromosome for inactivation is initiated from a single locus, the X inactivation center (XIC) on the X (reviewed in [2]). This locus encodes the non-coding *Xist* RNA whose accumulation is necessary and sufficient to mark the chromosome for inactivation [3]. In the worm *Caenorhabditis elegans,* gene expression from both X chromosomes in hermaphrodites is reduced to about half when compared to expression of the single X chromosomes in males (reviewed in [4]). This is achieved by the binding of a multi-subunit dosage compensation complex (DCC) along the entire length of both X chromosomes. In this case, the selection of the chromosomes is not due to a single locus, but is thought to involve multiple recognition sites for the DCC [5,6]. In the fly *Drosophila melanogaster,* dosage compensation is also achieved by a large ribonucleoprotein complex (reviewed in [7–9]). The *Drosophila* DCC, however, is recruited to the single male X chromosome and leads to an approximate two-fold increase in transcription of many X-linked genes [10,11]. The selection of the X in flies uses a system with similarities to both mechanisms mentioned above, relying in part on the expression and accumulation of two non-coding RNAs from the X and in part on the presence of specific recruitment sites distinguishing the X from other chromosomes [12,13].

The DCC of D. melanogaster contains at least five proteins and one of two non-coding RNAs that are all essential for male survival. The three male-specific lethal proteins MSL1 [14], MSL2 [15], and MSL3 [16] are thought to provide structural and regulatory functions [17,18] whereas the males absent on the first (MOF[19]) and maleless (MLE [20]) proteins contribute acetyltransferase and helicase activity, respectively. The function of the two non-coding RNAs, *roX1* (RNA on the X) and *roX2,* is so far unknown. However, all the structural components and both enzymatic activities, as well as at least one RNA, are required for proper targeting of the DCC to the male X and its distribution over the entire chromosome [16,19,21–23]. MSL2 is the only component that is strictly male specific [24], and its presence in the nucleus leads to the stabilization of other complex components and the accumulation of the DCC on the X chromosome [25]. In females, the translation of MSL2 mRNA is inhibited by the Sex-lethal protein (SXL) and the complex does not form [26,27]. However, unregulated ectopic expression of MSL2 leads to DCC formation and binding to both X chromosomes [25,27,28]. This fact has allowed detailed study of the mechanism of DCC targeting and distribution in *Drosophila*.

**Figure 1:**
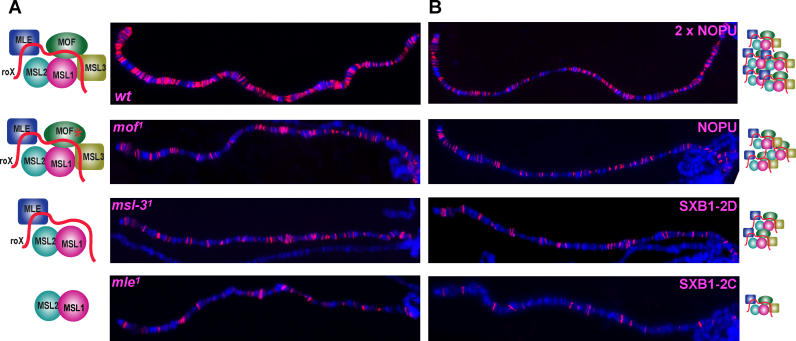
The *Drosophila* X Chromosome Contains a Hierarchy of DCC Binding Sites Polytene chromosomes from *Drosophila* larvae showing the DNA stained in blue and anti-MSL1 staining in red. (A) The DCC binds in a defined pattern to the X chromosome in wild-type males (top panel). Mutations in *mof, msl-3,* and *mle* lead to partial or non-functioning complexes (cartoons) binding to a subset of sites. Anti-MSL-1 staining of X chromosomes from females expressing MSL2 and homozygous for the *mof^1^, msl-3^1^,* and *mle^1^* mutations are shown. (B) The same hierarchy of sites can be seen in females expressing different concentrations of MSL2. The NOPU and SXB1–2 constructs give rise to decreasing levels of MSL2 expression [27], and this leads to the recruitment of the DCC to decreasing numbers of sites on both X chromosomes. These sites are the same as those found in the mutant backgrounds described in (A) [28]. Females homozygous for the NOPU construct (top panel) have a DCC binding pattern quite similar to wild-type males, lacking only a few sites.

Immunostaining of polytene chromosomes from the salivary glands of *Drosophila* larvae allows the visualization of proteins on interphase chromatin and has been used extensively in the study of dosage compensation [29]. The binding pattern of the DCC is well defined, being restricted to interbands on the male X chromosome [28] (Figure 1A). Mutations in DCC components change this binding pattern. If MSL1 or MSL2 are mutated, the entire complex is lost from the X chromosome. Mutations in the remaining components lead to binding of partial complexes to a defined subset of sites [22,28,30]. This is illustrated in Figure 1A, which shows the distribution of MSL1 on polytene chromosomes of female larvae expressing MSL2 ectopically and carrying mutations in *mle, msl-3,* and *mof*. The subset of binding sites remaining in the *msl-3* background was originally termed “chromatin entry sites” [31]. A longstanding model for how the DCC is localized to the X assumed that these distinctive loci served as primary recruitment sites for the DCC from which the complex distributes to less-defined chromatin by spreading in *cis* [32]. Lowering the levels of MSL2 available in the nucleus also leads to binding of the DCC to a reduced set of sites [27,28]. MSL2-expression constructs with mutations in the 5′ and/or 3′ UTR that make them less sensitive to regulation by SXL (SXB-1, NOPU), can be used to express different levels of MSL2 protein in females (Figure 1B, [27]). As previously noted [28], the subset of sites visible in these females matches those visible in females carrying *msl* mutations .

The identification and characterization of two specific binding sites for the DCC within the *roX1* and *roX2* genes provided support for the “recruitment and spreading” model [31,33,34]. The *roX1* and *roX2* genes are part of the restricted set of sites that appear in the *msl-3^1^* mutant [32]. When *roX* genes were inserted into autosomes via P element transformation, the DCC was recruited to the insertion site both in wild-type and mutant backgrounds, suggesting that these genes contain “entry sites” for the complex [31]. Detailed studies led to the identification of a 110 base pair (bp) DNA binding element within male-specific DNAseI hypersensitive sites in both *roX1* and *roX2* [35]. Importantly, the DCC could occasionally be seen to spread from the ectopic *roX* loci to neighboring autosomal chromatin [31]. The spreading was characterized by DCC binding close to the insertion sites on polytene chromosomes. Further studies revealed that the spreading could be expanded by raising the levels of DCC present in the cell or reducing the number of *roX* loci [36].

The “entry-site and spreading” model appeared attractive because of its similarity to current views of how mammalian X inactivation is achieved. However, the generality of the model was challenged by more recent studies, which showed that recruitment of the DCC is not a property of a small set of entry sites, as many large X-derived fragments were able to recruit the complex to ectopic autosomal sites [37,38]. Furthermore, characterization of a third DCC binding site at polytene band 18D10 revealed only minimal spreading in rare cases [37]. No spreading was observed from X to autosome translocations, even when they contained a *roX* gene [38]. In addition, when pieces of autosomes were translocated to the X, the DCC did not bind to the translocated chromatin, but stayed specific to X derived chromatin [37,38]. Collectively, these results lead to the proposition of an alternative model for the recruitment of the DCC to the X not involving spreading, but rather involving a large number of specific binding sites with varying affinities for the complex [38,39]. In this model, each site of DCC binding on the X involves specific recognition and only depends on the concentration of the complex available. Studies of DCC binding in females expressing varying levels of MSL2, discussed above, support the concept of sites with different affinities [28].

Clearly, evaluation and refinement of current concepts for DCC recruitment requires analysis of a larger number of DCC binding sites. In this study we have used chromatin immunoprecipitation (ChIP) to identify a number of new DCC binding fragments (DBFs) from the X and characterized them in vivo. Among them are sites that are bound by the DCC even in *msl* mutant backgrounds (novel “chromatin entry sites” by the old nomenclature). We show that the identified fragments have different abilities to recruit the DCC when inserted into autosomes. By varying complex levels, we gained support for the existence of sites with different affinities for the complex on the X. Rare cases of secondary binding sites close to high-affinity inserts appear not to be due to non-specific spreading of the complex along the chromatin fiber, but rather to recognition of low-affinity binding sites nearby. In addition, we identified pairs of sequence elements that are common between the new high-affinity DBFs and previously characterized sites, and found that some of these are modestly enriched on the X.

## Results

### Identification of DCC Binding Fragments Using ChIP

**Figure 2:**
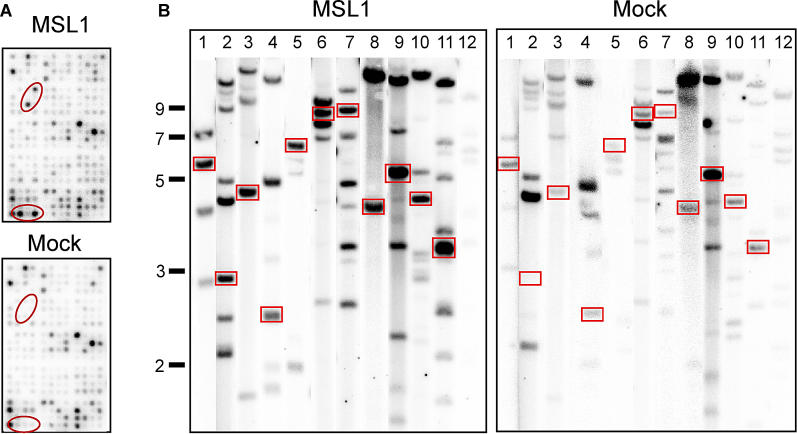
Identification of New DCC Binding Fragments Using Chromatin IP (A) Subsection of genomic P1 phage clone filter, showing 120 clones out of a total of 9,216, hybridized with MSL1 ChIP probe (upper panel), then stripped and re-hybridized to the mock IP control (lower panel). Two examples of clones showing MSL1 enrichment chosen for further analysis are indicated by red circles. Note that clones are always spotted in duplicate on the membrane. (B) Southern blots of digested P1 clone DNA hybridized to MSL1 ChIP (left panel) or mock ChIP (right panel). Red boxes highlight the bands enriched in the MSL1 ChIP chosen for cloning, known as DBF1–DBF14. Size in kilobases is indicated to the left of the figure. Lane order and restriction digests are as follows: Lane 1, DBF1 (BglII); 2, DBF3 (XhoI); 3, DBF5 (XhoI); 4, DBF6 (ApaLI); 5, DBF7 (BamHI); 6, DBF9 (XhoI); 7, DBF10 (EcoRI); 8, DBF11 (ApaLI); 9, DBF12 (EcoRI); 10, DBF13 (EcoRI); 11, DBF14 (EcoRI); and 12, autosomal DNA control P1 clone (EcoRI). Note that clones DBF2, DBF4, and DBF8 (false positives) have been omitted from this figure. P1 phage clone identifiers are listed in the Materials and Methods section.

To identify DNA fragments associated with the DCC, we performed ChIP from mixed-sex *Drosophila* embryos using anti-MSL1 antibodies. Because the DCC is not present in female embryos, these are expected to contribute only background-level signals in the ChIP. Labeled DNA recovered from the ChIP experiments was hybridized to a commercial membrane (previously available from Genome Systems) containing 9,216 bacteriophage P1 clones [40,41] with an average size of 85 kb, theoretically representing approximately six times genome coverage. Visual comparison of MSL1 versus mock ChIP signals revealed several clones that were enriched in the MSL1 ChIP (Figure 2A). Strikingly, all clones mapped to the X chromosome.

With the aim of identifying strongly hybridizing restriction fragments for cloning and further mapping, we prepared bacteriophage DNA from each of the clones. The MSL1 ChIP probes were used to identify strongly hybridizing restriction fragments ranging from 1 to 8.5 kb within the P1 clones (Figure 2B). In cases in which no single band could be identified as the strongest site of hybridization, two bands were selected. Hybridization with an MSL3 ChIP probe to the digests produced a similar pattern, verifying the enrichment of the DCC on these fragments (data not shown). The selected restriction fragments were cloned and subjected to a third round of hybridization. Quantification of the MSL1 ChIP signals compared to the mock signals allowed us to select a single candidate fragment from each P1 clone. The 5′ and 3′ ends of the clones were sequenced to allow precise localization of the fragments on the X chromosome and the projected full-length sequences obtained from release 4 of the *Drosophila* genomic sequence. The identified fragments, named “DCC binding fragment” (DBF) 1–14, are listed in Table 1 with associated accession numbers. All the fragments contain genes or parts of genes (exons and introns) and some contain intergenic regions. DBF14 overlaps with the previously identified high-affinity DCC binding site at 18D [37] and was not analyzed further in this study.

**Table 1:**
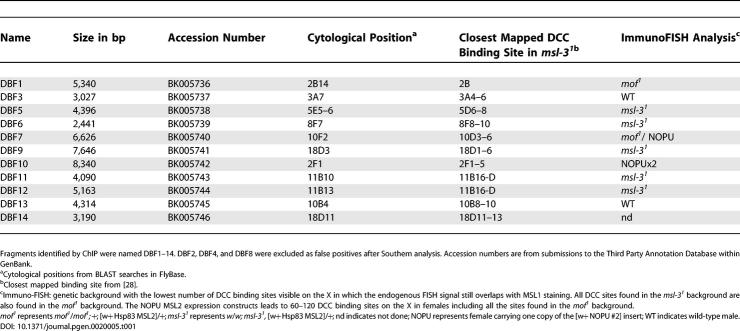
DCC Binding Fragments Identified by ChIP

### MSL1 Binding to Endogenous Loci of DCC Binding Fragments

We first wanted to explore if the DBFs identified using ChIP were bound by the DCC in vivo, and specifically if they could be part of the subset of sites seen in the *msl-3^1^* mutant. The fact that one of the fragments, DBF14, had already been shown to overlap with the DCC binding site at 18D was encouraging [37]. In addition, when the cytological positions of the DBFs were compared with the mapped positions of high-affinity sites [28], several were found to putatively overlap with such sites (Table 1).

**Figure 3:**
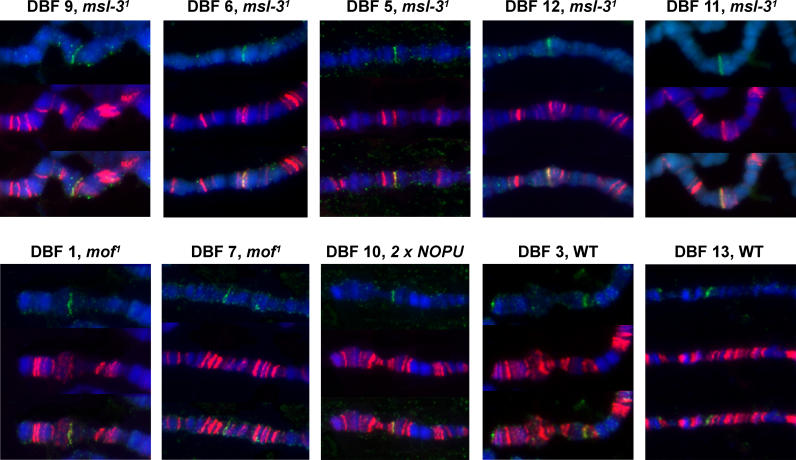
The Endogenous Loci of the DCC Binding Fragments Overlap with MSL1 In Vivo Immuno-FISH experiments using the identified DBFs as probes (FISH signals in green) and staining with anti-MSL1 antibodies to visualize DCC binding (in red). Each panel consists of images of the FISH signal (top), the anti-MSL1 signal (middle), and the merge (bottom). The FISH signals from DBF9, DBF6, DBF5, and DBF12 overlap with the anti-MSL1 signals in the *msl-3^1^* mutant background (*w*/*w; msl-3^1^,* [w+ Hsp83 MSL2]). DBF11 overlaps partially with MSL1 staining in this background. DBF1 and DBF7 overlap with weak anti-MSL signals in the *mof^1^* mutant background (*mof^1^*/*mof^1^;* +; [w+ Hsp83 MSL2]/+). FISH signals from the endogenous loci of DBF3, DBF10, and DBF13 do not overlap with MSL1 staining in either the *msl-3^1^*or *mof^1^* background. However, DBF10 does overlap with MSL1 staining in females carrying two copies of the NOPU insert (2 × NOPU, Figure1), whereas DBF3 and DBF13 overlap with MSL staining in wild-type males (WT).

To investigate if the fragments indeed overlapped with MSL1 binding on polytene chromosomes, we carried out combined immunofluorescence and fluorescence in situ hybridization (immuno-FISH) experiments using the anti-MSL1 antibody and part of the DBF sequences as probes. Initial experiments performed in the *msl-3^1^* background revealed that the FISH signals for five of the ten fragments (DBF12, DBF11, DBF9, DBF6, and DBF5) did indeed overlap with anti-MSL1 signals (Figure 3). The overlap of the signals was confirmed by confocal microscopy (data not shown). Two of the DCC binding fragments, DBF12 and DBF11, are located only 20 kb apart within the same gene. Confocal analysis revealed that the FISH signal for DBF12 overlapped almost completely with the MSL1 signal, whereas the signal for DBF11 overlapped only minimally (data not shown). The remainder of the identified fragments did not overlap with significant anti-MSL1 signal in the *msl-3^1^* background. We therefore repeated the immuno-FISH in other genetic backgrounds in which additional DCC binding sites are present on the X (see Figure 1). We found that FISH signals for DBF1 and DBF7 overlapped with MSL1 staining in the *mof^1^* background and in females carrying one copy of the NOPU insert (although the MSL1 signals are often weak) (Figure 3). The endogenous DBF10 overlapped with MSL1 staining in females carrying two copies of the NOPU insert, whereas DBF13 and DBF3 only overlapped with DCC binding sites in wild-type males (Figure 3). In summary, the results from the immuno-FISH experiments (summarized in Table 1) indicated that the fragments identified by ChIP were all very close to or coincident with in vivo DCC binding sites and hence good candidates for DCC binding determinants. Furthermore, the set of fragments contains representatives of different classes of DCC binding determinants, as defined by MSL1 binding in different genetic backgrounds.

### In Vivo Analysis of DCC Binding Fragments

**Figure 4:**
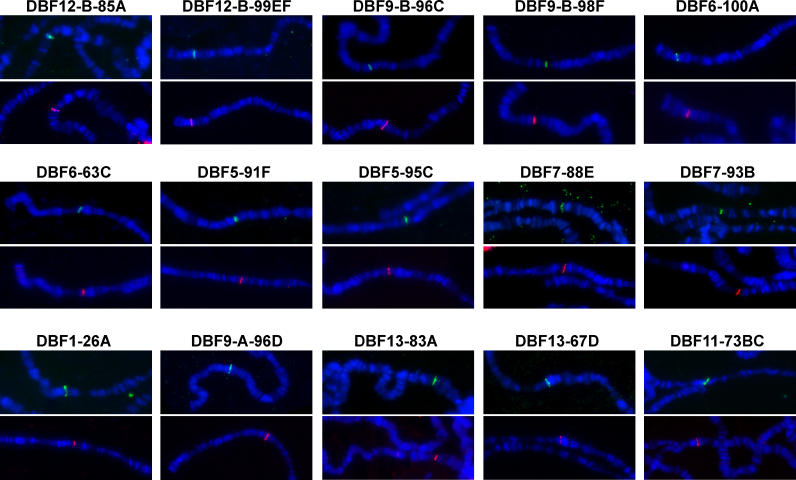
Recruitment of the DCC to DBF Insertions in Wild-Type Males Polytene chromosomes with FISH signals in green and anti-MSL1 signals in red showing examples of DBF insertions recruiting the DCC in wild-type males. The cytological position of DBF inserts was estimated from FISH experiments (upper image in each panel). Recruitment of the DCC was demonstrated with immunoflourescence using anti-MSL1 antibodies (lower image in each panel). Examples from all DBF inserts that showed recruitment in wild-type males are shown. A summary of the complete analysis can be found in Table 2.

It is possible that the DNA fragments found to be associated with the DCC by ChIP do not represent recruitment sites for the DCC, but rather represent the distribution of active complex on the X chromosome. In order to investigate if the new DBFs were able to recruit the DCC in vivo, we employed the same assay as was used in the analysis of the *roX1, roX2,* and 18D sites [31,37]. The ten DBFs were cloned into the pCasper4 P-element vector for generation of transgenic flies. Preliminary analysis of the DBF sequences had revealed that a number of them contained OPA repeats [42]. To investigate a possible role for OPA repeats in DCC binding in vivo, DBF12 was split to yield a 5′ construct containing the OPA sequences (DBF12-A) and a 3′ construct devoid of such repeats (DBF12-B). Due to its large size, DBF9 was also cloned in two halves, generating DBF9-A and DBF9-B. For each construct, several different insertions of the P element on the second and/or third chromosomes were selected. The insertion sites were mapped by DNA FISH using the mini-white gene as a probe (Figure 4). Recruitment of the DCC to the inserts was analyzed by anti-MSL1 immunostaining on polytene chromosomes. It had previously been shown that the presence of MSL1 entails the presence of the whole DCC [31]. For each DBF, we analyzed between three and five different inserts in order to minimize bias from position effects. For each insert in each genetic background, a total of 50–100 nuclei from two or three different individuals were examined. It was immediately clear that the different DBF insertions possessed different DCC recruitment abilities. Variation could be observed in the signal intensity of the anti-MSL1 staining compared to the signals on the X as well as the number of nuclei in which the signal could be observed. Typically, strong signals were observed in a high percentage of nuclei, whereas weak signals could only be observed in a subset of nuclei. In addition, different insertions of the same DBF showed different recruitment abilities indicating that they were subject to position effects.

**Table 2:**
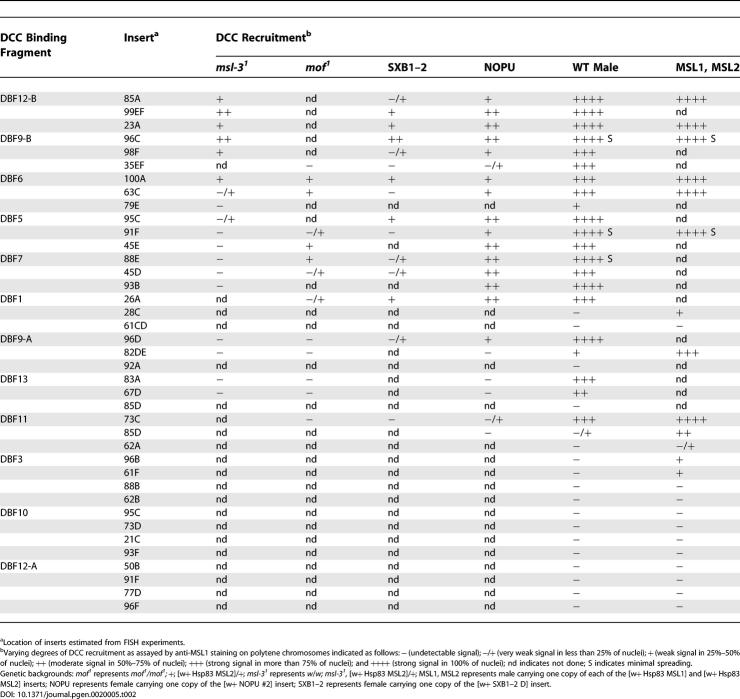
Summary of In Vivo Analysis of DCC Binding Fragments

Recruitment of the DCC to autosomal insertions of DBF constructs was first investigated in wild-type males. For nine out of the eleven DBFs, anti-MSL1 signals could be detected for at least one insert. DBF5, DBF6, DBF7, DBF12-B, and DBF9-B had strong recruitment ability at all insertion sites, whereas DBF1, DBF9-A, DBF11, and DBF13 had variable recruitment ability (Table 2). These initial results indicated that the enrichment of the DBF sequences in the MSL1 ChIP indeed reflects an in vivo association of the DCC with most of these sequences. No anti-MSL1 signal could be detected at any inserts of DBF3 and DBF10. These fragments are also among the weakest DCC interaction sites in their native X chromosomal context (see Figure 3). Inserts of DBF12-A were also unable to recruit the DCC, excluding a connection between OPA repeats and DCC binding. The variability in the recruitment of the DCC indicates that the identified fragments have different affinities for the complex.

### Recruitment of the DCC at Varying Complex Concentrations

**Figure 5:**
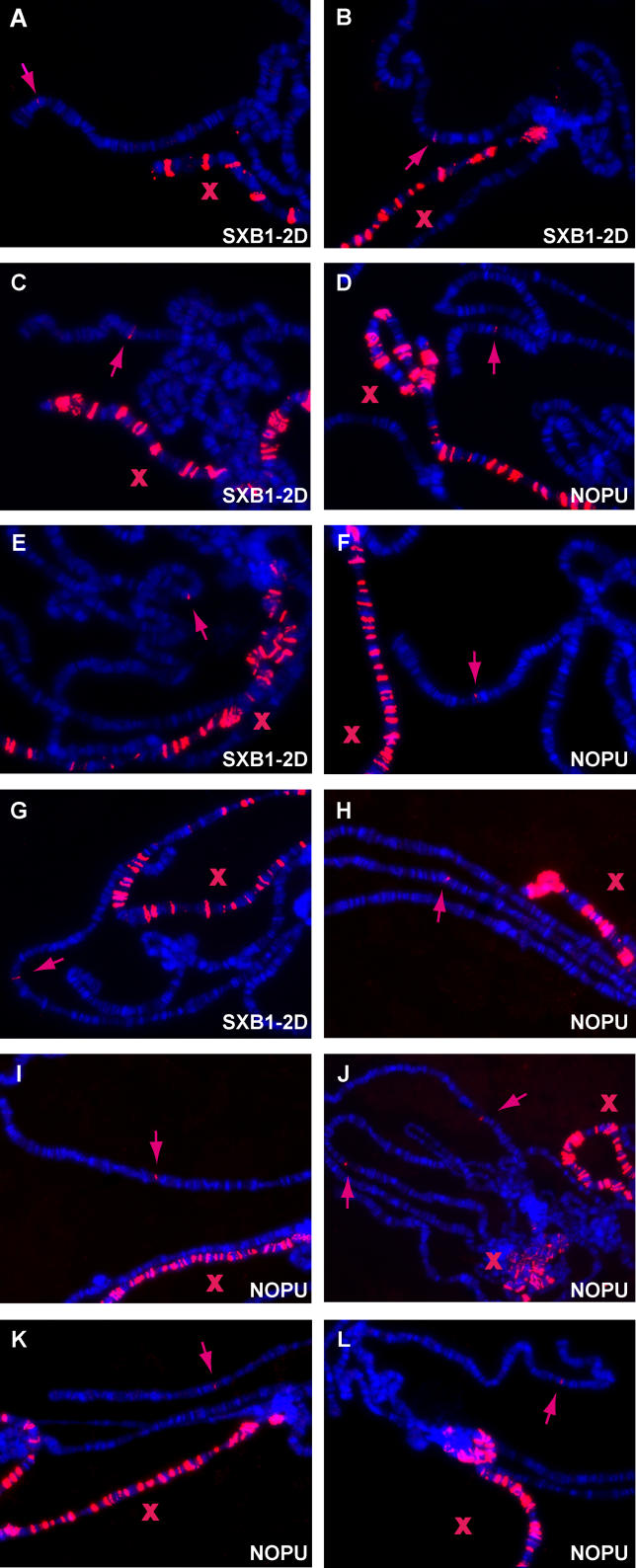
Several DCC Binding Fragments Can Recruit the Complex When MSL2 Levels Are Limiting Examples of DCC recruitment to DBF inserts in females carrying one copy of the SXB1–2 or NOPU MSL2 expression constructs (see Figure 1). Anti-MSL1 staining is shown in red and DNA staining in blue. Arrows indicate the position of the inserts and X indicates the X chromosome. Inserts DBF12-99EF (A), DBF12-85A (B), DBF9-96C (C), DBF6-100A (E), and DBF5-95C (G) recruit the DCC at lower levels of MSL2 in the SXB1–2 background, whereas inserts DBF9B-98F (D), DBF6-63C (F), DBF5-91F (H), DBF7-88E (I), DBF7-93B (J), DBF1-26A (K), and DBF9A-96D (L) recruit the complex in the NOPU background. For a summary of the complete results, see Table 2.

Investigation of differences in affinity between DBFs requires that the levels of intact complex be varied. As discussed in the introduction, expression of varying MSL2 levels in females leads to corresponding levels of intact DCC, which associates with both X chromosomes (Figure 1). We first investigated the ability of the ten DBFs to recruit the DCC in females carrying one copy of the SXB1-2D MSL2-expression construct. Due to lower than wild-type MSL2 levels only 30–60 DCC binding sites are visible on the X chromosomes of these females (Figure 1, [27]). Those fragments that gave rise to the most robust binding in wild-type males were also able to recruit the DCC in this background, albeit less efficiently (Figure 5). Insertions of DBF12, DBF9-B, and DBF5 showed intermediate anti-MSL1 signals, whereas insertions of DBF6, DBF7, and DBF1 showed only very weak and occasional signals. None of the other fragments seemed to recruit the DCC at this concentration of complex (Table 2). Next we analyzed recruitment ability in females carrying one copy of the NOPU MSL2 construct. In these females, the DCC binds to between 60–120 sites on the X, reflecting higher levels of MSL2 expression (Figure 1,[27]). Under these conditions the recruitment potential of DBF6, DBF7, and DBF1 was enhanced (Table 2). In addition, one insert of both DBF9-A and DBF11 showed weak anti-MSL1 signals (Figure 5). These results provide further evidence that the DBFs we isolated have different affinities for the DCC in vivo.

**Figure 6:**
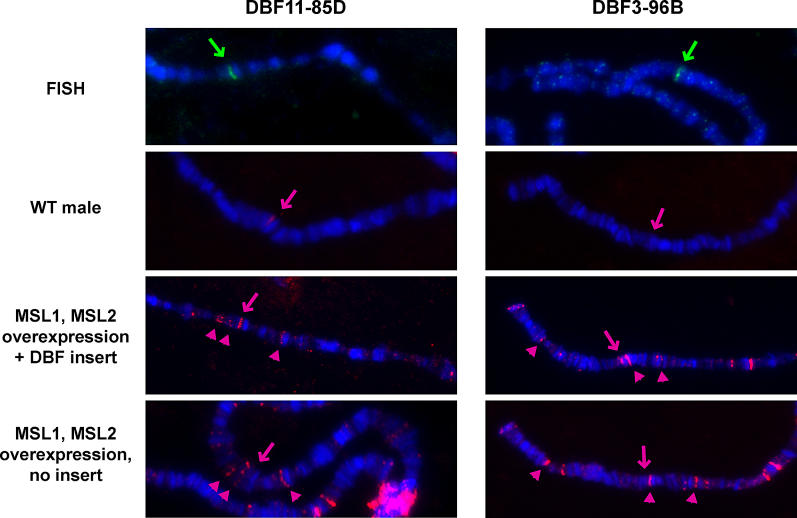
Increasing the Levels of the DCC Increases Recruitment to Low-Affinity Binding Fragments Polytene chromosomes showing FISH signals (top panels, green) and anti-MSL1 staining (red) in the genetic backgrounds indicated. Arrows indicate the position of the inserts and arrowheads indicate autosomal sites of DCC binding in the MSL1 and MSL2 over-expression background. Recruitment of the DCC to the DBF11-85D insert is enhanced when the levels of DCC are increased by over-expression of MSL1 and MSL2 compared to wild-type males. The DBF3-96B insert does not recruit the DCC in wild-type males, but recruitment can be seen when MSL1 and MSL2 are over-expressed.

None of the autosomal inserts of DBF3 or DBF10 were able to recruit the DCC in wild-type males. In addition, some inserts of DBFs with moderate recruitment ability (i.e., DBF11 and DBF13) were also negative for MSL1 binding in vivo (Table 2). We wanted to investigate if increasing the concentration of the DCC in the nucleus would allow DCC binding to such sites. Over-expressing MSL1 and MSL2 in male flies raises the levels of the DCC leading to increased binding to the X, which disrupts the morphology of this chromosome [43]. In addition, the association of the DCC with many autosomal sites becomes visible [28]. We found that DBF inserts with weak recruitment potential showed more robust binding of the DCC under conditions of MSL1 and MSL2 over-expression (Figure 6 and Table 2). In addition, some inserts that were not bound in wild-type males, such as inserts of DBF3, recruited the DCC when MSL1 and MSL2 were over-expressed (Figure 6, Table 2). This suggests that DBF3 has very low affinity for the complex, whereas DBF10 and DBF12-A have no detectable affinity in this assay. However, we cannot exclude that critical DCC binding determinants were lost during the selection and subcloning of these fragments from the original P1 clones.

### Recruitment of Partial Complexes

**Figure 7:**
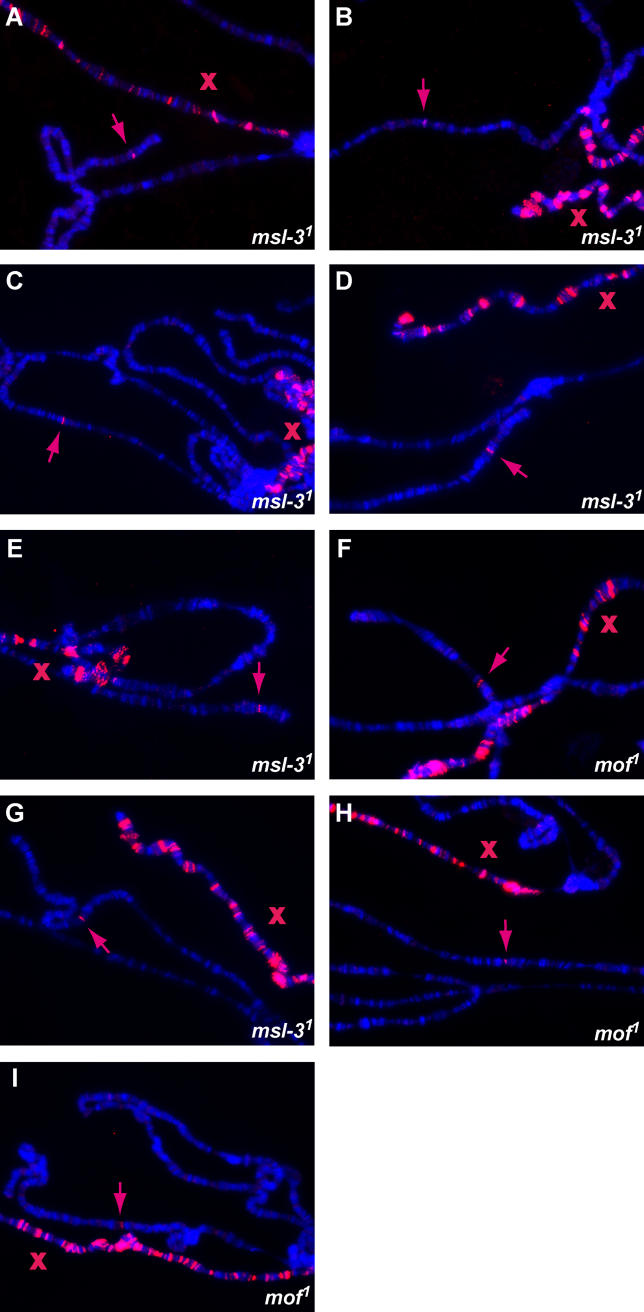
High-Affinity DCC Binding Fragments Recruit Partial and Inactive Complexes in *msl-3^1^* and *mof^1^* Mutants Examples of DCC recruitment to DBF inserts in females expressing MSL2 and carrying mutations in *msl-3^1^* (*w*/*w; msl-3^1^,* [w+ Hsp83 MSL2]/+) or *mof^1^* (*mof^1^*/*mof^1^;* +; [w+ Hsp83 MSL2]/+). Anti-MSL1 staining is shown in red and DNA staining in blue. Arrows indicate the position of the inserts and X indicates the X chromosome. Inserts DBF12-B-99EF (A), DBF12-B-85A (B), DBF9-B-96C (C), DBF9B-98F (D), DBF6-100A (E), and DBF5-95C (G) recruit partial complexes in the *msl-3^1^* mutant. Inserts DBF6-63C (F), DBF5-91F (H), and DBF7-88E (I) recruit inactive complexes in the *mof^1^* mutant background.

To investigate the ability of the DBFs to recruit partial and non-functional DCC complexes, we looked for anti-MSL1 staining in the *msl* mutant backgrounds. The analysis was done in females expressing MSL2 and homozygous for the *msl-3^1^* or *mof^1^* mutations. All the inserts that showed recruitment in wild-type males were tested for recruitment of complexes lacking MSL3. From the initial immuno-FISH analysis, we knew that at least four of the endogenous DBF loci overlap with DCC binding sites in this background. Indeed, DBF12-B, DBF9-B, and DBF6 were able to consistently recruit partial DCCs (Figure 7) and, therefore, qualify as novel “chromatin entry sites” in the old terminology. One insert of DBF5 also shows weak anti-MSL1 signals in the *msl-3^1^* background, so may be considered a fourth new entry site. The *mof^1^* mutation leads to the formation of a DCC that lacks the histone acetyltransferase activity. In terms of DCC recruitment to the X, the *mof^1^* mutation is less damaging than the *msl-3^1^* mutation (Figure 1). DBF insertions that recruit the DCC in the *msl-3^1^* background would therefore also be expected to recruit the DCC in a *mof^1^* mutant background, and this was demonstrated for a subset of inserts (Table 2). In addition, two inserts of DBF7 were able to recruit the DCC weakly in the *mof^1^* background (Figure 7). The binding fragments with low affinity for the DCC were not able to recruit partial or mutant complexes (Table 2). We conclude that the affinity of binding sites as judged from recruitment at different DCC levels correlates with their ability to attract partial or non-functional complexes.

### Additional Binding of the DCC Close to Ectopic High-Affinity Sites

**Figure 8:**
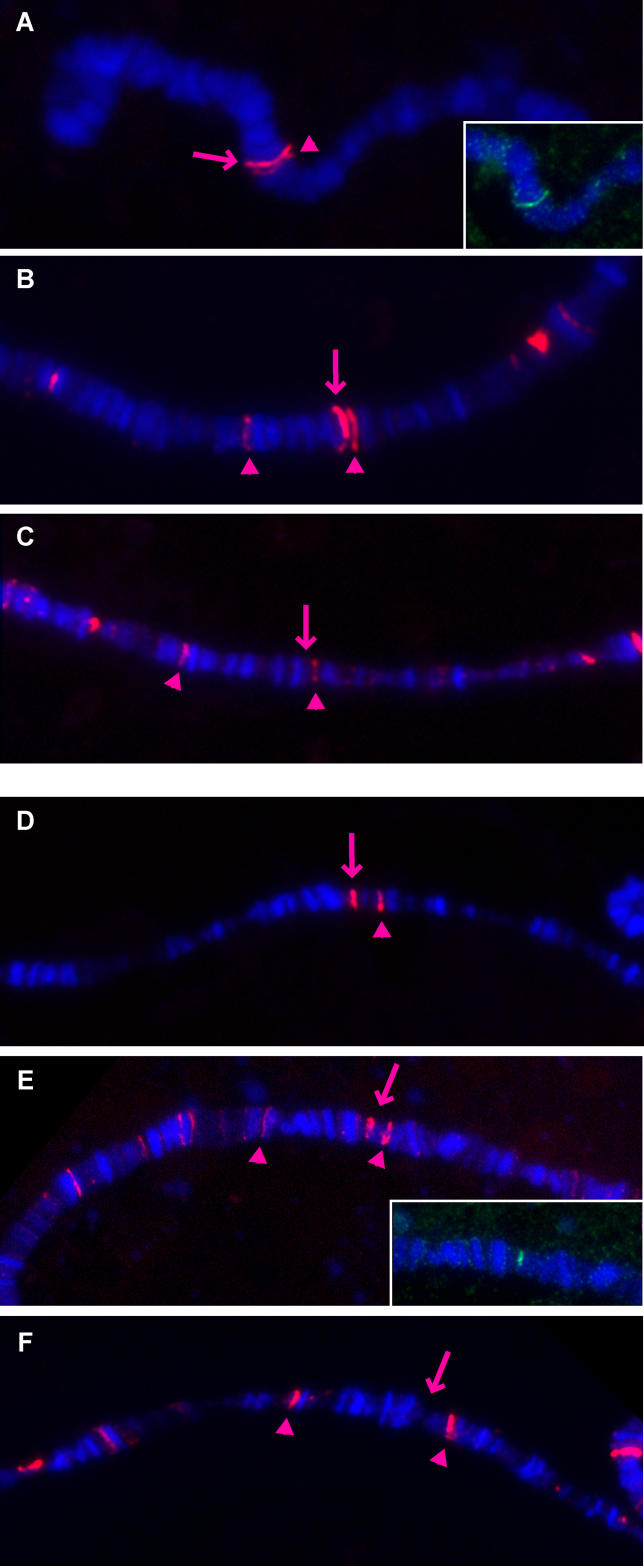
Minimal Spreading from High-Affinity Sites to Low-Affinity Autosomal Sites Examples of two inserts, DBF9-96C (A–C) and DBF5-91F (D–F), that cause occasional spreading of the DCC to one additional band next to the insertion site in wild-type males (A and D). When complex levels are increased by over-expression of MSL1 and MSL2, the secondary DCC binding sites appear as part of the set of autosomal binding sites for the complex (B and E) and are present even in the absence of the DBF insert (C and F). Anti-MSL1 staining is shown in red and DNA staining in blue. Small inserts in (A) and (E) show the FISH signals (green) corresponding to the P element insertion. Arrows indicate the position of the inserts and arrowheads indicate autosomal sites of DCC binding.

During our analysis of the DBFs in wild-type males, we observed rare cases of additional DCC binding close to inserts of the high-affinity fragments DBF12, DBF5, and DBF7 (Table 2 and Figure 5). This is reminiscent of the minimal “spreading” of the DCC observed from the *roX1* and *roX2* transgenes [31,36] and the more recently described 18D site [37]. Because more extensive spreading from *roX* transgenes (both in terms of number of additional binding sites and distance from the insertion) could be achieved by raising the levels of DCC [28,36], we investigated if we could induce spreading from other high-affinity sites by over-expressing MSL1 and MSL2. Even at increased DCC levels, we could not detect additional bands of DCC binding close to any inserts other than those already seen to support such additional binding events. For the three inserts that did show a secondary band in wild-type nuclei, this band could be detected more often if MSL1 and MSL2 were over-expressed (data not shown). However, the secondary bands associated with the DBF9-B-96C and DBF5-91F inserts could also be observed in controls over-expressing MSL1 and MSL2 in which the inserts were not present (Figure 8). Consequently, these additional bands belong to those autosomal sequences of lowest affinity that attract complex only if DCC concentrations are experimentally increased beyond physiological levels.

### Identification of Common Sequence Elements in DCC Binding Fragments

This study has lead to the identification of a number of new high-affinity binding fragments for the DCC. We explored whether it was possible to identify common elements between these and previously characterized fragments that may contribute to defining DCC binding sites. We considered DBF12-B, DBF9-B, DBF6, DBF5, and DBF7 to contain high-affinity sites as they produced consistent DCC recruitment in wild-type males as well as in *msl-3^1^* and/or *mof^1^* mutants. In addition, we included the full-length *roX1* and *roX2* sequences [44] and the 8.8 kb fragment of 18D [37]. DBF10 and two large autosomal regions from 2L and 3R represent fragments with no affinity for the DCC (no recruitment of the DCC on polytene chromosomes) and thus provided control sequences. Initial analysis suggested that there were no simple nucleotide sequences or “words” (6–10 bp) in common between all the high-affinity fragments that were also absent from the control fragments. It is, however, conceivable that the DCC binding sites consist of combinations or clusters of different words. In order to search for such combinations, we limited the search to words that were present in the *roX1* or *roX*2 DHS fragments [33,35] as these are the best-studied high-affinity sites. Using these words, we identified a set of “elements” present in at least six out of the eight candidate fragments that consist of families of 6–8 bp words related to each other by a defined allowance for mismatches (see Materials and Methods). None of these elements were absent in the control sequences.

In order to investigate possible clustering, we looked for pairs of elements found within a 5–200 bp window. We identified a large number of pairs present in at least six out of eight of the high-affinity fragments and absent in controls. Many of the pairs were very similar or overlapping, and by merging related pairs, we could generate a list of 61 pairs of elements with two or fewer mismatches between the individual words of each element. All of these pairs were absent in the control sequences and present in a larger number of high-affinity fragments (seven or eight out of eight) than the individual component pairs (a list of all 61 element pairs is available on request).

It would be expected that any element or combination of elements (such as a pair) involved in dosage compensation should be over-represented on the X chromosome compared to other chromosomes. We therefore individually compared the frequency of occurrence of each of the 61 element pairs on the X to the frequency on the five other chromosome arms. We found that 24 of the pairs were modestly, but significantly, enriched on the X compared to other chromosomes. Table S1 contains a list of the pairs with significant enrichment on the X, grouped together according to sequence similarities. For each pair, we also calculated the percentage of occurrence on the X out of total genomic occurrences. Compared to the percentage expected from a random distribution, all pairs occurred more often on the X than expected (Table S1). The X chromosome enrichment of most of the pairs may be explained by the presence of GAGA-related sequences in one of the elements. It has previously been shown that GA dinucleotide repeats are over-represented on the X [45,46]. It is interesting to note, however, that most of the pairs are found at appreciably higher frequency in the high-affinity fragments compared to the moderate- and low-affinity fragments DBF1, DBF9-A, DBF11, DBF13, and DBF3 (Table S1). Our results suggest that it is possible to identify pairs of short elements in common between most of the high affinity fragments, which are also over-represented on the X chromosome. However, the localization of these pairs is not exclusive to the X and therefore not sufficient to explain the X-specific localization of the DCC**.**

## Discussion

### DCC Binding Sites of Different Affinities

Using a ChIP strategy, we have identified several new DCC binding fragments and demonstrated that they possess a wide range of potential to recruit the DCC. Because the majority of the isolated candidate fragments co-map with endogenous DCC binding sites at the resolution afforded by staining of polytene chromosomes, we believe our ChIP selection procedure was appropriate. By tuning DCC levels in vivo, we came to conclude that the difference in recruitment ability is due to different affinity of the DCC for these fragments. At limiting concentrations of complex, only the sites of highest affinity are occupied. Conversely, at non-physiologically high concentrations of DCC, even “cryptic” binding sites on autosomes are recognized by the complex. This suggests, in concordance with previous observations [28], that selective interaction of the DCC with the X chromosome is a function of tightly controlled levels of complex components that are adjusted to assure interaction with binding sites of varying affinity clustered on the X, but insufficient to occupy cryptic sequences on autosomes. Our data are also in broad agreement with recent observations from Oh et al. [37] and Fagegaltier and Baker [38], who found that numerous sites on the X chromosomes contain DCC binding determinants. We now show that these determinants are not all equal, but represent a diverse set of DCC targets that differ by a wide range of affinities for the complex, as expected from a sequence determinant that became gradually enriched on the X chromosome during evolution.

The use of the term “chromatin entry sites” for the subset of DCC binding sites that are still occupied by partial complexes in the absence of MSL3 [31], implied that these sites were somehow qualitatively and perhaps functionally distinct from the remaining sites that only attract the intact complex. Although it is possible that not all DCC binding sites are functionally equivalent, our characterization of several new examples of both types of DCC binding sites rather supports the “affinities model” [28,38]. According to this model, “chromatin entry sites” are not qualitatively different from other sites, but only represent those sites with the highest affinity for the complex. A prediction from this model that is further substantiated by our results is that non-functional complexes that lack MSL3 or the acetyltransferase activity of MOF have lower affinity for target sites. Only those determinants with highest affinity for the DCC are able to recruit partial complexes in the absence of MSL3. Sites with slightly lower affinity are still able to recruit the complex in the *mof^1^* mutant. Because the interaction of the DCC with the X chromosome is thought to be largely mediated by MSL1 and MSL2 [47], it remains to be explored whether MSL3 and the acetylase activity of MOF affect the active concentration of MSL1 and MSL2 or lead instead to the adoption of a high-affinity conformation of the complex. Conversely, it remains to be seen if over-expression of MSL1 and MSL2 in the *msl-3^1^* and *mof^1^* mutants would allow partial complexes to bind additional sites. In this respect it is intriguing that the mutation of both *roX* RNAs, which is presumed to lead to incomplete and non-functional complexes, can be partially rescued by the over-expression of MSL1 and MSL2 [30,43].

### Distribution of the DCC

During our analysis of DCC recruitment to high-affinity sites inserted into autosomes of wild-type males, we observed an additional band of DCC binding close to the insertion site in three independent cases (one insert each of DBF9, DBF5, and DBF7). Such minimal and rare “spreading” has previously been observed for ectopic insertions of the 18D high-affinity site [37] and from *roX* transgenes in the wild-type male background [36]. Our study now reveals that these additional DCC binding sites are not a result of random spreading, but are most likely due to interaction of the DCC with one of the low-affinity sites on autosomes, which happened to reside close to the insertion site (Figure 8). These sites are usually only observed when the DCC concentrations are globally increased by over-expression of MSL1 and MSL2 [28]. Accordingly, we suggest that the autosomal insertion of a high-affinity DCC binding site leads to a local rise in complex concentration, which allows these low-affinity sites to be recognized by the DCC even in wild-type males. However, additional requirements must clearly be met to allow low-affinity sites to profit from local increases in complex concentration, as not all ectopic high-affinity sites support the phenomenon (I. K. Dahlsveen, unpublished data). Permissive conditions may include active transcription or the presence of specific epigenetic marks.

We envision that the clustering of DCC binding determinants of high and intermediate affinity on the X chromosome (combined with the transcription of the *roX* RNAs) elevates the concentration of the DCC within the X chromosomal territory and ensures the occupancy of lower-affinity sites in a context-dependent manner. This may explain the observation that autosomally derived transgenes often acquire dosage compensation. The transgenes may contain cryptic DCC binding determinants and may thus acquire binding if placed in the context of the X chromosomal territory. Conversely, an X chromosomal fragment that harbors only low-affinity sites may not be recognized if translocated to an autosomal context, and our fragment DBF3 may be an example for such a scenario. The presence of a large number of low-affinity sites may also contribute significantly to restricting the binding of the DCC to the X chromosome.

The term “spreading” has been used to describe the appearance of additional bands of DCC binding around autosomal insertions of *roX* cDNAs or fragments derived thereof [31,33]. However, extensive, long-range spreading from *roX* transgenes, which leads to the appearance of many ectopic DCC bands at greater distances from the insertion sites, only occurs under unusual conditions and depends on the transcription of the *roX* RNA rather than the DCC binding sites on DNA [36,48]. Long-range spreading of the complex also does not occur into autosomal chromatin translocated to the X chromosome [37,38]. We suggest that large translocations maintain their original chromosomal context (DCC enriched or not), and therefore no redistribution of DCC over the new chromosomal junction is observable at the resolution of the polytene chromosomes. Importantly, our study does not address the higher-resolution distribution of the DCC within a chromosomal band. It is possible that such a band contains many individual binding sites, also of varying affinity. At this resolution, the term “spreading” may characterize the local diffusion of the DCC from high- to low-affinity sites. Our study does not exclude this type of spreading, or indeed any other kind of complex distribution within a chromosomal band. High-resolution ChIP analyses will be necessary to resolve the detailed nature of DCC distribution.

### Defining DCC Binding Sites

Previously, only three high-affinity binding sites for DCC were known [35,37]. Our study identified nine more fragments, which encouraged investigation of common features within a larger pool. Interestingly, we find that all new DBFs map to gene-rich regions and either overlap with or lie close to essential genes. Three high-affinity fragments (DBF12, DBF9, and DBF6) reside entirely within genes. It is possible that specific recruitment sites, such as those inferred to reside within our DBFs, have been enriched in and around genes that require dosage compensation during evolution [8], and consequently, high-affinity sites may represent loci that are particularly dosage sensitive. Previous experiments indicated that the DCC tends to bind to the coding regions of genes [49], and it was suggested that this was linked to transcriptional activity [50]. Although recent observations suggest that transcriptional activity alone is not sufficient to attract DCC binding [51], it is possible that transcription influences DCC recruitment to specific sites. For example, high-affinity sites, which in our assays show consistent and strong recruitment of the DCC at many chromosomal positions, may not be influenced by transcription. However, sites with lower affinity and variable recruitment ability may profit from transcriptional activity. Developmental differences in transcriptional activity may therefore also explain the lack of DCC recruitment in salivary glands to fragments isolated by ChIP from embryos.

We have attempted to identify common sequence elements within previously characterized and new high-affinity DCC binding fragments and have uncovered a number of short sequence elements, whose clustering in combinations could contribute to DCC recruitment. Clearly, the importance of these elements remains to be tested experimentally. Previous analysis of the *roX* DCC binding sites identified a 110 bp sequence containing several blocks of conservation between *roX1* and *roX2* [35]. DCC binding was affected by mutation in several of the conserved blocks, indicating that DCC binding sites may be made up of combinations of shorter elements. We have started to look for such combinations by defining pairs of elements found within a 200 bp window in the high-affinity DCC binding fragments. Those pairs that are significantly enriched on the X chromosome compared to other chromosomes are listed in Table S1. Importantly, these X-enriched pairs often occur in multiple copies in the high-affinity fragments (not shown) and at higher frequencies compared to the lower-affinity fragments DBF9-A, DBF1, DBF11, DBF13, and DBF3 (Table S1). Nonetheless, there is no obvious correlation between the location of individual pairs on the X and any specific features such as predicted genes. We hypothesize that the elements that define these pairs (and other such elements that may have escaped our attention) correspond to building blocks of DCC binding sites. Accordingly, a DCC binding site of given affinity for the complex would not be determined by a unique DNA sequence, but by clustering of variable combinations of short, degenerate sequence motifs, as previously suggested [8]. Individual low-affinity binding sites may not be unique to the X, but their clustering on the X may contribute to high-affinity binding [52]. We already have indications that the DCC binds to several sites in close proximity. The two parts of DBF9, DBF9-A and DBF9-B, are both able to recruit the DCC, albeit with different affinity. The analysis of the 18D high-affinity fragment also suggested that multiple elements over 8.8 kb contribute to the binding of the complex [37].

**Figure 9:**
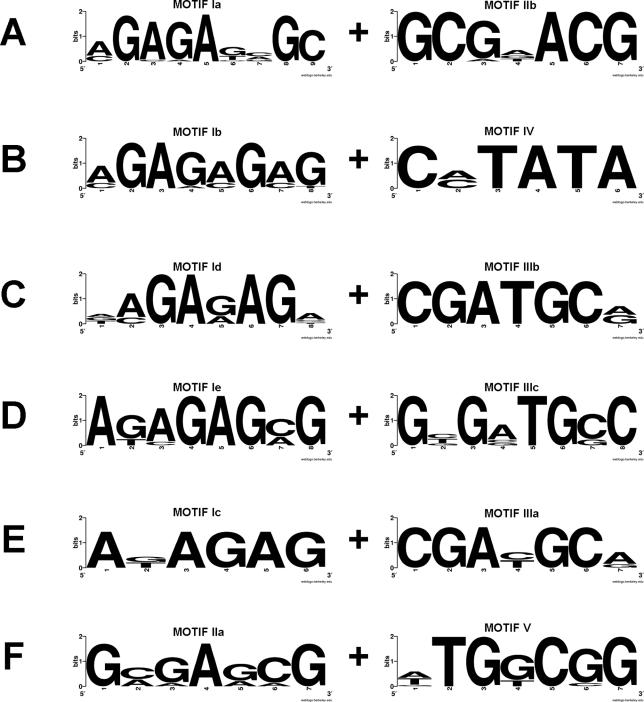
DNA Sequence Logos of Representative Motifs Putatively Involved in DCC Binding Logos (generated using the WebLogo software) represent degenerate motifs based on related pairs of elements from Table S1. The logos were generated by aligning all the words of related elements from specific pairs: (A) pairs 5, 6, 7, 8, 9, and 10; (B) pairs 11, 12, 13, and 14; (C) pairs 17, 18, 19, and 20; (D) 21, 22, and 23; (E) pairs 15 and 16; and (F) pairs 1, 2, and 3. Pairs 4 and 24 were not included. Motifs Ia–Ie all contain GAGA-related sequences. Motifs IIa and IIb as well as IIIa–IIIc are also related to each other.

In Table S1, we have ordered the pairs according to sequence similarity. Interestingly, a large family of elements contain GAGA-related motifs. Mutation of GAGA or CTCT motifs in the 110 bp *roX1*/*roX2* consensus severely affected DCC recruitment to that sequence, indicating that GAGA motifs are involved in DCC binding [35]. The fact that we find these elements enriched in several independently identified high-affinity fragments demonstrates the appropriateness of our algorithms. Besides elements with a clear relationship to GAGA motifs, we also noticed several other element families defined by sequence similarity (separated by broad horizontal bars in Table S1). In order to visualize the element families, the related words may be aligned such that sequence logos representing degenerate motifs can be derived using the WebLogo software (http://weblogo.cbr.nrc.ca). Pairs of representative motifs are shown in Figure 9, but the logos are less constrained than the original pairs of elements and therefore do not only represent pairs specifically enriched on the X. However, we consider it possible that some of these degenerate motifs may contribute to DCC binding sites. Evaluation of the contributions of these novel motifs to the targeting of the complex will require increased resolution analysis and systematic evaluation of candidate sequences in the in vivo recruitment assay.

### DCC–DNA Interactions

Our study suggests that high-affinity DCC binding sites are composed of variable combinations of clustered, degenerate sequence motifs. The degeneracy of the sequence motifs indicates that many individual elements may have low affinity. Therefore, the interaction of the DCC with each individual site should be in dynamic equilibrium. However, we recently observed by photobleaching techniques that the DCC components most likely involved in chromatin binding, MSL2 and MSL1, interact with the X chromosomal territory in cultured cells in an unusually stable manner [53], which is not compatible with binding equilibria involving off-rates that commonly characterize protein–DNA interactions. Several hypotheses can be formulated, whose evaluation may lead to resolution of this apparent contradiction. First, formation of higher-order structures involving many DCC components engaged in numerous simultaneous DNA interactions may lead to a trapping of the DCC within the X chromosome territory. Second, an initial sequence-directed targeting event may be followed by a stabilization of the interaction through positive reinforcement involving additional principles, such as epigenetic marks or a topological linkage (see [53] for discussion). Finally, we consider that the arrangement of the interphase genome in polytene chromosomes may differ in a relevant aspect from the more compact chromosomal territories of diploid cultured cells. Ultimately, the identification of the DNA-binding domains of DCC components and analysis of their mode of DNA interaction will be required to solve the targeting issue.

## Materials and Methods

### P1 clones and cloning of DBFs.

Bacteriophage P1 clone (sources of DBF clones) identifiers are as follows: DBF1 (DS00954); DBF2 (DS05757); DBF3 (DS02577); DBF4 (DS04038); DBF5 (DS00375); DBF6 (DS05338); DBF7 (DS00951); DBF8 (DS06397); DBF9 (DS09077); DBF10 (DS08342); DBF11 (DS08372); DBF12 (DS04200); DBF13 (DS01141); DBF14 (DS04283). Clones were obtained from the Berkeley Drosophila Genome Project (http://www.fruitfly.org) and maintained on LB agar containing 25 μg/ml kanamycin. During early to mid log-phase growth in liquid culture, P1 phage replication was induced by addition of 1 mM IPTG and cells harvested after a further 2–3 h. DNA was prepared using Qiagen Maxiprep kits (QIAGEN GmbH, Hilden, Germany). The genomic location of two clones had been previously mapped by in situ hybridization [40], seven had been partially sequenced [54], and short sequences were generated in our lab for the remaining clones.

For cloning of highly enriched fragments from the ChIP, P1 phage DNA was digested with restriction enzymes as detailed in Figure 2 and the DNA ligated with pBluescript SK (Stratagene, La Jolla, California, United States) digested with EcoRI (DBF10, DBF12, DBF13, and DBF14), XhoI (DBF3, DBF5. and DBF9) or BamHI (DBF7). In three cases, insert DNA was filled-in with Klenow polymerase prior to cloning into pBluescript SK digested with EcoRV (DBF1, DBF6, and DBF11). All clones were maintained in *Escherichia coli* XL-1 Blue (Stratagene).

DBF P-element clones were prepared by releasing inserts from DBF pBluescript subclones (using original cloning sites or flanking sites in the polylinker) and inserting into pCaSpeR4. In addition, DBF9 and DBF12 were each divided into two smaller sub-fragments and cloned into pCaSpeR4; DBF9-A (5.5 kb XhoI/BamHI fragment), DBF9-B (3.9 kb Asp718I/XhoI fragment), DBF12-A (3.0 kb EcoRI/Asp718I fragment), DBF12-B (2.6 kb XhoI/EcoRI fragment).

### Chromatin immunoprecipitation.

ChIPs were performed on chromatin prepared from 12- to 14-h-old mixed-sex embryos as described previously, including purification over a CsCl gradient [33]. Embryos were fixed in 4% formaldehyde at 18 °C for 15 min. Affinity-purified anti-MSL1 antibody (2 μl/ immunoprecipitation [IP]) was a gift from M. Kuroda. Following IP and reversal of cross-links, recovered DNA was resuspended in a final volume of 22 μl of H_2_O. Seven microliters of this DNA was incubated with Pfu polymerase, ligated to linker, and subjected to linker-mediated PCR prior to random priming for use as a probe in Southern hybridization.

### Southern transfer and hybridization.

Approximately 1 μg of bacteriophage P1 DNA per lane was digested with restriction enzyme and loaded on 0.8% agarose gels. Electrophoresis and Southern transfer was performed as described [10]. A total of 100 ng of LM-PCR amplified DNA from MSL1 or mock ChIP was labeled using the Megaprime labeling system (Amersham Biosciences, Little Chalfont, United Kingdom), purified over G-50 columns (Roche, Mannheim, Germany) and equal amounts of specific or mock IP, as determined by scintillation counting, used as a probe in Southern hybridization. Southern hybridization was performed as described [55]. Signals were quantitated using a Fuji FLA-3000 phospho-imager (Tokyo, Japan). Due to the presence of repetitive elements in some clones, subtracting mock IP signals from the MSL1 IP signal was used as an indicator of a clone's binding to MSL1.

### *Drosophila* genetics.

Flies were raised on standard cornmeal-agar-yeast medium (including 0.25% Nipagin) at 18 °C or 25 °C. All the stocks carrying MSL mutations and MSL2 expression constructs were kindly provided by M. Kuroda and R. Kelley. Alleles used in this study were: *msl-3^1^, mof^1^, msl-2^1^, mof^+t6.8^, msl-2^SXB1–2^, msl-2^NOPU^, msl-1^Hsp83.PC^,* and *msl-2^Hsp83.PK^*. To generate transgenic flies carrying DBF insertions, fragments were cloned into the pP[CaSpeR-4] vector and the resulting P elements injected into *w^1118^* or *y^1^w^1118^* stocks that also served as the wild-type background. Several different insertions on the second and third chromosomes were generated for each construct.

In order to analyze the DBF insertions in females expressing different levels of MSL2, homozygous inserts on the second or third chromosome (or heterozygous insert on the third/TM6) were crossed to *w; msl-2^1^ cn;* [w+ SXB1-2D] or *w;* [w+ NOPU #2] and female larvae selected for analysis. To analyze insertions in males over-expressing MSL1 and MSL2, inserts were crossed to *w/Y; msl-2^1^ cn*/+; [w+ Hsp83 MSL1] [w+ Hsp83 MSL2]/TM6 males and non-tubby males selected for analysis. The analysis in the *msl-3^1^* and *mof^1^* mutant background involved generating stocks carrying both the DBF inserts ([w+ DBF]) and *msl* alleles and the insert for the expression of MSL2. Inserts on the third chromosome were recombined with the *msl-3^1^* allele and non-tubby female larvae from the following cross selected: *w*/Y; *msl-3^1^* [w+ DBF]/TM6 x *w*/*w; msl-3^1^* [w+ Hsp83 MSL2]. For inserts on the second chromosome, non-tubby female larvae from the cross *w*/Y; [w+ DBF]; *msl-3^1^* [w+ Hsp83 MSL2]/TM6 x *w*/*w; msl-3^1^* [w+ Hsp83 MSL2] were selected. To generate the *mof^1^* mutant background, the DBF inserts and the Hsp83 MSL2 were combined with the *mof^1^* allele. For inserts on the third chromosome, non-tubby female larvae from the cross *mof^1^*/Y; *mof^+t6.8^*/+; [w+ DBF]/TM6 x *mof^1^*/*mof^1^;* +; [w+ Hsp83 MSL2]/(TM6) were selected. The absence of the *mof^+t6.8^* rescue construct and the *mof* phenotype was confirmed after staining with anti-MSL1 antibodies. For inserts on the second chromosome, female larvae from the cross *mof^1^*/Y; *mof^+t6.8^*/[w+ DBF];+ x *mof^1^*/*mof^1^;* +; [w+ Hsp83 MSL2]/(TM6) were selected. In this case, the absence of the *mof^+t6.8^* rescue construct also selected for the presence of the insert.

### FISH, immunostaining, and immuno-FISH to polytene chromosomes.

Probes for DNA FISH were prepared with the Prime-It II kit (Stratagene) using up to 500 ng of DNA template and a mixture of biotinylated ATP (biotin14dATP) and dATP. For immunostaining, one of two affinity-purified rabbit anti-MSL1 antibodies kindly provided by M. Kuroda [31] and E. Schulze [56] were used at a dilution of 1:200 and 1:400, respectively. DNA was stained with Hoechst 33258.

Preparation of polytene chromosomes was carried out as described [57] with these modifications: For FISH, glands were fixed in 45% acetic acid for 5.5 min; for immunostaining and immuno-FISH, glands were fixed in 1.85% formaldehyde (from frozen stocks) in 45% acetic acid for 10 min and 6 min, respectively. After freezing, slides for FISH were washed in 95% ethanol, air dried, and stored at room temperature; slides for immunostaining and immuno-FISH were washed in PBS and used immediately, or in the case of immunostaining only, stored in 100% methanol for up to 1 wk.

For FISH, slides were pre-treated by incubation in 2× SSC at 65 °C for 45–60 min, dehydrated in 70% ethanol (2 × 5 min) and 95% ethanol (2 × 5 min), and air dried. Denaturation was done in 0.07 M NaOH for 3 min followed by several washes in 2× SSC and dehydration as above. After precipitation, probes were resuspended in 100–150 μl of hybridization buffer (50% formamide, 10% dextran sulphate, 0.6 mg/ml denatured salmon sperm DNA, 2× SSC) and denatured at 95 °C for 6 min. Slides were incubated overnight with 15–20 μl of hybridization solution at 37 °C in a humid chamber. Following washing with 2× SSC at 42°C (3 × 5 min) and PBS, 0.1% triton at 22 °C (2 × 5 min), detection was performed using Cy2-labelled Streptavidin (1:100) in PBS, 0.1% triton, 1% BSA for 60 min at 22 °C. Slides were washed in PBS (3 × 5 min), stained with Hoechst (0.4 μg/ml for 2 min) and mounted in 50% glycerol, 0.1 M n-propyl-gallate in PBS.

For immunostaining, slides were blocked in PBS, 0.1% triton, 1% BSA for 60 min and incubated overnight at 4 °C with the primary antibody (in PBS, 0.1% triton, 1% BSA). After washing in PBS and PBS, 0.1% triton, 1% BSA, detection was performed using Cy3 conjugated anti-rabbit antibody for 60 min at 22 °C. Slides were washed, stained with Hoechst, and mounted as above.

For immuno-FISH, the immunostaining was performed first as described above. The quality of the immunostaining and the correct phenotype were confirmed by microscopy. The cover slips were washed off in PBS, and after an additional washing step, the slides were transferred to 2× SSC and incubated at 70 °C for a maximum of 45 min and the FISH performed as described above. This protocol will preserve the anti-MSL1 staining, although the signal becomes much weaker.

### Bioinformatics.

References for sequences used are as follows: roX1-c3 [58]; roX1 DHS: bp 981-1281 of roX1-c3; roX2–78.13 [44]; roX2 DHS: bp 983-1252 of roX2–78.13; 18D10 [37]; control 2L: 2L:1556825..1609283; and control 3R: 3R:15497200..15524289. Accession numbers for DBF sequences can be found in Table 1. Initial search for words, *N* = 6–10, was performed by complete listing of all nucleotide combinations in *roX1* and *roX2* DHS fragments. “Families” of words were created by allowing all possible mismatches defined as follows: 6 bp and 7 bp words were allowed one mismatch, 8 bp and 9 bp words were allowed two, and 10 bp words were allowed three mismatches. The high-affinity fragments were then searched for all possible words and word families. Those words or word families present in at least six out of eight fragments were called “elements.” The list of elements was used to identify 657 element pairs with the following specifications: Elements of a pair have to reside within a 5 to 200 bp window, be present in at least six out of eight fragments, and be absent from control sequences. Pairs related to each other were identified and merged by superimposition. Superimpositions of pairs were calculated from direct and diagonal superimpositions of the original 657 pairs, allowing variation in individual elements of up to two positions. Where the word lengths within a family differed, the number of mismatches was calculated as the minimal number of mismatches between the shortest word and all possible subsequences (of equal lengths to that of the shortest word) within the longer words. Only superimpositions that were present in a higher number of high-affinity sites than the individual components they are made of were included in the final list of 61 pairs.

The frequency of occurrences of the pairs of elements in the genome was calculated using sequences from release 3.2 of the Berkeley Drosophila Genome Project (BDGP) (downloaded from http://feb2005.archive.ensembl.org/Download; Drosophila_melanogaster.BDGP3.2.1.feb.dna.chromosome.2L.fa.gz, etc.). The number of occurrences *(N)* of each pair was determined for each of the six chromosome arms: X, 2L, 2R, 3L, 3R, and 4, and normalized by the length of the chromosome (L). The standard deviation is equal to the square root of *N* normalized by L. The enrichment on the X is significant when the fraction *N*/L is more than three standard deviations higher compared to the fraction for another chromosome arm. Enrichment on the X over all five of the other chromosome arms was defined as overall significant enrichment. The percentage of occurrences on the X was calculated as the percentage of total occurrences on all chromosome arms. The frequency of occurrence in high- versus moderate- and low-affinity elements was calculated by determining the total number of occurrences in each set of fragments divided by the total number of kilobases.

DNA sequence logos were made online using the WebLogo software (version 2.8.1) at http://weblogo.cbr.nrc.ca. All words in related elements from the specified pairs presented in Table S1 were aligned to generate the logos.

## Supporting Information

Table S1 Pairs of Elements in High-Affinity DBFs with Significant Enrichment on the X (92 KB DOC)

### Accession Numbers

The FlyBase (http://flybase.bio.indiana.edu/genes) accession numbers for the alleles, genes, and gene products discussed in this paper are MLE (FBgn0002774), MOF (FBgn0014340), *mof^+t6.8^* (FBal0059999), *mof^1^* (FBal0059998), MSL1 (FBgn0005617), *msl-1^Hsp83.PC^* (FBal0093826), MSL2 (FBgn0005616), *msl-2^1^* (FBal0012561), *msl-2^Hsp83.PK^* (FBal0044201), *msl-2^NOPU^* (FBal0061031), *msl-2^SXB1–2^* (FBal0061029), MSL3 (FBgn0002775), *msl-3^1^* (FBal0012357), *roX1* (FBgn0019661), *roX2* (FBgn0019660). The FlyBase (http://flybase.bio.indiana.edu/transposons) accession number for the pP[CaSpeR-4] P element vector is (FBmc0000178).

The GenBank (http://www.ncbi.nlm.nih.gov/Genbank) accession number for rox2–78.13 is U85981. The GenBank Third Party Annotation accession numbers for the DCC binding fragments discussed in this paper are DBF1 (BK005736), DBF3 (BK005737), DBF5 (BK005738), DBF6 (BK005739), DBF7 (BK005740), DBF9 (BK005741), DBF10 (BK005742), DBF11 (BK005743), DBF12 (BK005744), DBF13 (BK005745), and DBF14 (BK005746).

## Abbreviations

bp: base pair
ChIP: chromatin immunoprecipitation
DBF: dosage compensation complex (DCC) binding fragments
DCC: dosage compensation complex
FISH: fluorescence in situ hybridization
immuno-FISH: combined immunofluorescence and fluorescence in situ hybridization
IP: immunoprecipitation
