# Supplementary material for: Targeting Determinants of Dosage Compensation in Drosophila
Source: PLoS Genet. 2006 Feb 3;2(2):e5. doi: 10.1371/journal.pgen.0020005 (PMC1359073; doi:10.1371/journal.pgen.0020005)
Supplement: Supplementary file 1 [file pgen.0020005.st001.doc]

**Supplementary table1.**

**Pairs of elements in high affinity DBFs with significant enrichment on the X.**

| **Pair**  **#** | **Element 1** | **Element 2** | **% on X^1^** | **Frequency in high affinity fragments^2^** | **Frequency in**  **low affinity fragments^3^** |
| --- | --- | --- | --- | --- | --- |
| 1 | ATGGCG  TTGTCG  TTGGCG | GCGAGCG  GAGAGCG | 21,5 | 0,48 | 0,18 |
| **2** | TTGTCG  TTGGCG  ATGGCCG  ATGGCGG | GCGAGCG  GAGAGCG  GAGAGAG | 23,5 | 0,84 | 0,18 |
| 3 | TGGCGG  ATGGCGG  GTGGCGG | GCGAGCG  GCGAACG  GCAAACG | 22,4 | 0,69 | 0,49 |
| 4 | GATCGA  GATCGC  GCTCGC | GCGGACG  GCGAACG | 23,4 | 0,36 | 0,27 |
| 5 | AGAGCG  AGATCG  CGAGCG | GCGGACG  GCGAACG | 23,1 | 0,48 | 0,22 |
| 6 | AGAGCG  AGATCG | GCGTACG  GCGGACG  GCGAACG | 23,1 | 0,45 | 0,22 |
| 7 | GAGACA  GAGAGA  CGAAAGA  CGAGA­­­GA | GCGAACG  GCAAACG | 23 | 0,72 | 0,36 |
| 8 | AGAGAGAG  AGAGAGCG  AGAGATGG | GCGAAC  GCGGAC  GCGTAC | 26,6 | 0,63 | 0,05 |
| 9 | GAGAGCG  GAGAGAG  AGAGAGCG  AGAGATGG | GCGAAC  GCGGAC  GCGTAC | 24 | 1.01 | 0.05 |
| 10 | GAGAGA  GAGACGGC  GAGATCGC  GAGATGGC | GCGAAC  GCGTAC | 23,4 | 1,01 | 0,05 |
| 11 | GAGCGAG  GAGAGAG  AGAGAGAG  AGAGAGCG | CATATA  CCTATA | 28,2 | 0,92 | 0 |
| 12 | GAGCGAG  GAGAGAG  AGAGAGAT  AGAGAGCG  CGAGAGAG | CATATA  CCTATA | 28,5 | 1,22 | 0 |
| 13 | GAGCGAG  GAGAGAG  AGAGAGCG  CGAGAGAG  AGAAAGAG | CATATA  CCTATA | 28,7 | 1,19 | 0 |
| 14 | GAGCGAG  GAGAGAG  AAAGAGAG  AGAGAGCG  CGAGAGAG | CATATA  CCTATA | 28,5 | 1,13 | 0 |
| 15 | AGAGAG  ATAGAG | CGACGC  CGACGCA  CGATGCA | 22,5 | 0,98 | 0 |
| 16 | AGAGAG  ATAGAG | CGACGC  CGATGCA  CGATGCG | 22,8 | 1,19 | 0 |
| 17 | AGAGAG  CGAAAG  CGAGAG | CGATGCA  CGATGCG | 21,7 | 0,87 | 0,05 |
| 18 | GAAAGA  AGAGAGT  AGAGAGA  AGAGAGC | CGATGCA  CGATGCG | 21,8 | 0,81 | 0,05 |
| 19 | GAAAGA  AAGAGAG  TAGAGAG  GAGAGAG | CGATGCA  CGATGCG | 22,3 | 0,84 | 0,05 |
| 20 | GAGAGG  GAAAGA  GAGAGA | CGATGCA  CGATGCG | 21,7 | 0,87 | 0,09 |
| 21 | TAGAGCG  AGAGAGAG  AGAGAGCG | GATGCC  GATGGC | 28 | 0,66 | 0,45 |
| 22 | GCGAGCG  AGAGAGAG  AGAGAGCG | CGATGC  CGGTGC  GGATGC | 26,7 | 1,13 | 0,36 |
| 23 | TAGAGCG  GAGAGCG  GAGAGAG | GTGCTG  GTGATG | 24,3 | 0,48 | 0,36 |
| 24 | AACGCAAG  ATCGAAAG | TCGATG  GCGATG  GAGATG | 21,8 | 0,33 | 0,13 |

Only pairs with significant enrichment (above 3 standard deviations) on the X compared to all other chromosome arms are shown. A pair is defined as two elements found within a 5-200 bp window in at least 7 out of 8 high affinity fragments and not in control sequences. Elements are families of words related by up to two mismatches.

Broad horizontal bars separate related elements.

**1** The percentage of occurrences on the X was calculated as the percentage of total occurrences on all chromosome arms. If distribution is random, 18,7% of occurrences are expected to be on the X.

**2** Frequency of occurrence per kb of the pair in all eight high affinity fragments (roX1, roX2, 18D, DBF12, DBF9, DBF6, DBF5 and DBF7).

**3** Frequency of occurrence per kb of the pair in moderate and low affinity fragments (DBF9A, DBF1, DBF11, DBF13 and DBF3).
